# Validation of a simplified procedure for convenient and rapid quantification of reduced and oxidized glutathione in human plasma by liquid chromatography tandem mass spectrometry analysis

**DOI:** 10.1002/bmc.4854

**Published:** 2020-06-28

**Authors:** Addison C. Enomoto, Erik Schneider, Toni McKinnon, Howard Goldfine, Mark A. Levy

**Affiliations:** ^1^ Research and Development, USANA Health Sciences Inc Salt Lake City UT USA

**Keywords:** clinical analysis, glutathione, GSH, GSSG, sample processing

## Abstract

Endogenous glutathione (GSH) and glutathione disulfide (GSSG) status is highly sensitive to oxidative conditions and have broad application as a surrogate indicator of redox status *in vivo.* Established methods for GSH and GSSG quantification in whole blood display limited utility in human plasma, where GSH and GSSG levels are ~3–4 orders of magnitude below those observed in whole blood. This study presents simplified sample processing and analytical LC–MS/MS approaches exhibiting the sensitivity and accuracy required to measure GSH and GSSG concentrations in human plasma samples, which after 5‐fold dilution to suppress matrix interferences range from 200 to 500 nm (GSH) and 5–30 nm (GSSG). The utility of the methods reported herein is demonstrated by assay performance and validation parameters which indicate good sensitivity [lower limits of quantitation of 4.99 nm (GSH) and 3.65 nm (GSSG), and high assay precision (intra‐assay CVs 3.6 and 1.9%, and inter‐assay CVs of 7.0 and 2.8% for GSH and GSSG, respectively). These methods also exhibited exceptional recovery of analyte‐spiked plasma samples (98.0 ± 7.64% for GSH and 98.5 ± 12.7% for GSSG). Good sample stability at −80°C was evident for GSH for up to 55 weeks and GSSG for up to 46 weeks, with average CVs <15 and <10%, respectively.

**Abbreviations**
GSH: reduced glutathione
GSH–NEM: glutathione–*N*‐ethylmaleimide
GSSG: oxidized glutathione (disulfide)
NEM: *N*‐ethylmaleimide
rcf: relative centrifugal force
ROS: reactive oxidative species
TCA: trichloroacetic acid

## 1 INTRODUCTION

Glutathione (GSH) is a tripeptide of glutamate, cysteine and glycine that serves as a central endogenous antioxidant that is critical for protection against oxidative stress induced by reactive oxidative species (ROS) (Asensi et al., 1998). In direct ROS‐neutralizing reactions, GSH is oxidized and converted from the free thiol form to glutathione disulfide (GSSG), leading to a decreased ratio of GSH to GSSG. Notably, measures of endogenous GSH and GSSG, and the ratio between GSH and GSSG, are regarded as valuable markers of oxidative status in tissues (Giustarini, Dalle‐Donne, Milzani, & Rossi, 2011), cells (Lu, 2013), biological fluids (Smith, Dunnett, & Mills, 1995) and, more recently, subcellular domains (Chen et al., 2017; Fernández‐Checa et al., 1997).

Although the concentration of, and the ratio between, GSH and GSSG within various cells and tissues have demonstrated relationships between oxidative status and specific disease states (Ballatori et al., 2009; Fernández‐Checa et al., 1997; Giustarini et al., 2011; Lu, 2013), the use of these indicators for the evaluation of redox status in human plasma has previously been limited by a wide range of factors (Claeson, Gouveia‐Figueira, Stenlund, & Johansson, 2019; Forgacsova et al., 2019). In particular, the relatively low concentration of GSH and GSSG in plasma (i.e. relative to other biological sample types) (Jones et al., 1998), as well as the poor sensitivity of traditional assay methods (Camera & Picardo, 2002) has presented a major challenge in terms of accurately and precisely quantifying GSH and GSSG in plasma at physiological concentrations in clinical research. In addition, methods such as HPLC–UV and HPLC–fluorescent spectroscopy that are currently utilized to detect GSH and GSSG at lower concentration ranges typically require laborious processing procedures and technically challenging preparation techniques (e.g. chemical derivatization) in order to ensure reliable measurements (Jones et al., 1998). Finally, many methods used to quantify GSH and GSSG are limited to applications utilizing specific sample types [i.e. whole blood or red blood cell (RBC) lysate] that are incompatible with plasma samples (Giustarini et al., 2011; Giustarini, Dalle‐Donne, Milzani, Fanti, & Rossi, 2013; Squellerio et al., 2012).

In addition to the methodological limitations noted above, numerous physico‐chemical factors affect GSH and GSSG stability, including sample pH as well as the composition of buffers and extraction reagents, which prevents the acquisition of reliable estimates of GSH and GSSG in many biological samples (Nishiyama & Kuninori, 1992), including human plasma (Jones et al., 1998). GSH is also susceptible to artifactual oxidations, which can markedly change both GSH and GSSG estimates *ex vivo*, thereby leading to misrepresentations of endogenous redox states (Giustarini et al., 2011; Jones et al., 1998; Nishiyama & Kuninori, 1992). Moreover, GSH and GSSG are susceptible to degradation and chemical modifications by proteolytic and phase II metabolic enzymes (e.g. *γ*‐glutamyltranspeptidases and glutathione‐*s*‐transferases) and glutathione reductases that can alter GSH and GSSG concentrations within samples during processing and storage (Jones et al., 1998; Lu, 2013). Notably, although certain protective groups have been utilized to stabilize GSH and GSSG levels in samples through the formation of thiol‐masked adducts, these “protected” derivatives are also sensitive to subtle fluctuations in pH and temperature and therefore do not unequivocally ensure accurate detection and quantification of GSH and GSSG (Giustarini et al., 2013; Nishiyama & Kuninori, 1992; Roosild et al., 2010).

In spite of the many factors that confound the assessment of GSH and GSSG concentrations in plasma, several groups have published methods to measure glutathione levels in animal as well as human plasma (Claeson et al., 2019; Forgacsova et al., 2019; Jones et al., 1998). However, technical limitations, particularly with regard to human plasma analysis of GSH and GSSG, limit their application in clinical research. In the current study, we describe a simplified plasma processing procedure along with convenient LC–MS/MS methods that provides a simple, convenient, accurate and precise method to quantify GSH and GSSG in human plasma sample stored for up to 55 weeks and 46 weeks, respectively. These attributes make this assay well suited for clinical research in which long‐term storage is often a necessity, and subtle changes in GSH or GSSG levels in plasma or serum are indicative of changes in health status, as has been observed, for example, in disease states such as diabetes (Costagliola et al., 1990), cystic fibrosis (Roum, Buhl, McElvaney, Borok, & Crystal, 2017) and HIV (Borges‐Santos, Moreto, Pereira, Ming‐Yu, & Burini, 2012), as well as in aging (Jones, Mody, Carlson, Lynn, & Sternberg, 2002).

## 2 EXPERIMENTAL

### 2.1 Reagents and buffer preparations

Reagent‐grade GSSG, *N*‐ethylmaleimide(NEM) and mono‐ and dibasic potassium phosphate (KH_2_PO_4_ and K_2_HPO_4_, respectively) were purchased from Sigma‐Aldrich(Milwaukee, WI, USA). Primary grade (P) reduced GSH reference standard was from ChromaDex (Irvine, CA, USA). Internal standards (IS) of glutathione ammonium salt‐d_5_ (GSH‐d_5_) and GSSG (GSSG‐^13^C_4_^15^N_2_) were from Toronto Research Chemicals (Toronto, Ontario, Canada) and Santa Cruz Biotechnology (Dallas, TX, USA), respectively. Reference standards for GSH and GSSG were ≥97% pure. Ultrapure (type 1) water purified using a Synergy UV‐R purification system (Millipore, Bedford, MA, USA) was used for all aqueous preparations detailed in the procedures described herein.

NEM stock solutions of 50 and 100 mm were prepared in type 1 water and then stored as single‐use aliquots at −20°C until use. Solutions of 0.2 m monobasic and dibasic potassium phosphate (KH_2_PO_4_ and K_2_HPO_4_, respectively) were prepared in water and stored for up to 2 weeks at 4°C until use. A solution of 0.2 m potassium phosphate buffer (PB) at pH 6.5 was prepared from KH_2_PO_4_ and K_2_HPO_4_ stock solutions and stored for up to 1 week at 4°C. Assay buffer was prepared fresh daily by diluting 5 vols of 0.2 m PB stock solution with 4 vols water and 1 vol of 50 mm NEM stock solution producing a working solution of 0.1 m PB containing 5 mm NEM used to prepare calibration standards and dilute plasma samples and included as a blank control in LC–MS/MS analyses of GSSG and GSH–NEM.

### 2.2 Preparation of standard stock solutions

A solution of 25 mg/ml GSH was prepared by dissolution of 25 mg GSH in water to a volume of 1 ml. The 25 mg/ml GSH solution (738 μl) was added to 7.5 ml of 0.2 m PB, 5.262 ml of water and 1.5 ml of fresh 100 mm NEM prepared in water providing 15 ml of 4 mm solution of ethylsuccinimido‐*S*‐glutathione (hereafter referred to as GSH–NEM), standard stock solution. The GSH–NEM stock solution was distributed into single‐use aliquots and stored at −80°C until use. A stock solution of 10 mm GSSG standard was prepared in 0.05 m potassium phosphate buffer and stored at −20°C in single use aliquots until use. Stock solutions of the internal standards, GSH‐d_5_–NEM (hereafter referred to as GSH–NEM–IS) for GSH and GSSG–^13^C_4_^15^N_2_ (hereafter referred to as GSSG–IS) for GSSG were prepared in 0.1 m PB at concentrations of 100 and 10 μm, respectively, and then stored in single‐use aliquots at −80°C until use. GSH–NEM, GSH–NEM–IS, GSSG and GSSG–IS working standards and calibration standards were prepared daily in assay buffer (0.1 m PB with 5 mm NEM) from stock solutions as described above.

### 2.3 Sample collection, plasma processing and storage

Human venous blood samples were drawn into 10 ml BD Vacutainer® collection tubes (366,401, BD Life Sciences) containing K_2_‐EDTA (lavender cap) by a certified clinician. Samples were processed immediately following collection as follows: vacutainers containing human blood samples were centrifuged at 1200 rcf for 12 min at 4°C immediately following collection to separate plasma. After centrifugation vacutainers were placed on ice and plasma was collected and transferred in 500 μl aliquots to 1.5 ml microcentrifuge tubes containing 50 μl of 50 mm NEM using a repeating pipet. Aliquots were vortexed briefly (~30 s) then stored at −80°C until use.

To prepare NEM‐treated plasma for LC–MS/MS analyses, frozen aliquots were thawed and immediately centrifuged at 20,000 rcf for 20 min at 4°C. Plasma supernatant (100 μl) was transferred to a 1.5 ml microcentrifuge tube, treated with 50 μl of 0.2 μmGSSG–IS for GSSG measurement, or 10 μmGSH–NEM–IS for GSH–NEM measurement, and brought to 450 μl with 0.1 m PB and 5 mm NEM. Samples were then treated with 50 μl of 100% (w/v) trichloroacetic acid (TCA), vortexed briefly then placed on ice for 5 min to facilitate precipitation of plasma proteins. Thereafter, samples were centrifuged at 20,000 rcf for 20 min at 4°C to separate the precipitate from the sample; 300 μl of supernatant from each sample was transferred to a new microcentrifuge tube and centrifuged once more at 20,000 rcf for 20 min at 4°C. Sample supernatant (200 μl) was then transferred to an 11 mm, 250 μl sample vial (C4011‐13, Thermo Scientific), sealed with an 11 mm aluminum crimp cap with PTFE/red rubber septa and placed into an Agilent Technologies 1,260 Infinity HiP ALS (G1367E) autosampler for LC–MS/MS analysis.

### 2.4 LC–MS/MS analysis of GSH–NEM and GSSG

Glutathione LC–MS/MS analyses were performed on an Agilent Technologies 1,260 Infinity System (Agilent Technologies, Santa Clara, CA, USA) consisting of an autosampler (G1367E), pump (G1311B) and column compartment (G1316A). Separation of analyte was achieved via isocratic elution using an Inertsil OD3 C_18_ column (150 × 4.6 mm, 3.0 μm; GL Sciences) and mobile phase of 15% 2‐propanol, 84.97% water and 0.03% formic acid for GSH–NEM analysis, and an Agilent XDB C_18_ column (50 × 4.6 mm, 1.8 μm; Agilent Technologies, Santa Clara, CA, USA) with a mobile phase of 99.97% water and 0.03% formic acid used for GSSG analysis. The flow rate was set to 0.5 ml/min with injection volumes of 20 μl (GSH–NEM) and 5 μl (GSSG). GSH–NEM ions and fragments were detected using an Agilent Technologies 6,460 triple quadrupole mass spectrometer (Agilent Technologies, Santa Clara, CA, USA) with electrospray ionization (ESI) in positive ion detection mode. GSH–NEM and GSSG were monitored by multiple reaction monitoring of ions at *m*/*z* 433.3 → 158.1 and *m*/*z* 613.6 → 231.1, respectively. Stable isotopes of reduced and glutathione oxidized glutathione (GSH–NEM–IS and GSSG–IS, respectively) were monitored at *m*/*z* 438.3 → 158.1 and *m*/*z* 619.6 → 231.1. Instrument parameters for GSH–NEM and GSSG and their respective internal standards were as follows: collision energy = 37 and capillary voltage = 3000 V. Nitrogen was used for the nebulizer and drying gas. Nebulizer pressure was 60 psi (~414 kPa), drying gas temperature was 350°C with a 12 L/min flow rate and the MS heater temperature was 100°C. LC–MS/MS data was acquired at 4.83 cycles/s and 207 ms/cycle with Agilent Technologies Mass Hunter software. Analyte concentrations obtained from the LC–MS/MS analysis were multiplied by 5 to account for plasma dilution during sample preparation.

### 2.5 Method validation

Assay specificity was evaluated via comparison of quantifier ion retention times in buffer vs. plasma and by comparing qualifier–quantifier ion ratios in buffer and in plasma using multiple reaction monitoring. Specifically, the retention time of the quantifier ion of the analyte of interest (i.e. *m*/*z* 158.1 for GSH–NEM or *m*/*z* 231.1 for GSSG) in buffer was compared with the retention time of the same ion in plasma. A difference in retention time of <6 s was considered as confirmatory. In order to compare qualifier–quantifier ion ratios, standards of GSH–NEM and GSSG were prepared in 0.1 m PB to identify parent ions in positive ion mode with an *m*/*z* ratio of 433.3 and 613.6 corresponding to GSH–NEM and GSSG, respectively. Product ion fragment abundance was then used to identify and calculate the ratios of two qualifier ions for each analyte (i.e. *m*/*z* 113.0, 84.1 for GSH–NEM; *m*/*z* 176.7, 178.7 for GSSG) to the quantifier ion for GSH–NEM and GSSG. These steps were then repeated in plasma samples. The assay was considered to be specific if the accuracy tolerance was within ±20% based on the following formula:

$$\text{Accuracy tolerance}\;\left(\%\right)=\left(1-\left[\frac{\text{mean buffer qualifier}–\text{quantifier ratio}}{\text{mean plasma qualifier}–\text{quantifier ratio}}\right]\right)\times 100\%$$

Calibration curve linearity was validated for LC–MS/MS analyses by preparing GSH–NEM calibration standards in 0.1 m PB containing 5 mm NEM at concentrations of 15.6, 31.3, 62.5, 125, 250, 500, 1000 and 2000 nm or GSSG calibration standards in 0.1 m PB containing 5 mm NEM at concentrations of 1.95, 3.91, 7.81, 15.6, 31.3, 62.5, 125, 250 and 500 nm. Note that, for all analysis, a known amount of internal standard was added to the calibration standards. The integrated peak area, or area under the curve (AUC) of the internal standard of the analyte of interest (i.e. GSH–NEM–IS for GSH–NEM or GSSG–IS for GSSG) was then used to calibrate or normalize the AUC of the analyte of interest in all calculations of analyte concentration. GSH–NEM and GSSG lower limits of detection (LLOD) and quantitation (LLOQ) were determined by identifying the lowest concentration of analyte detected in LC–MS/MS analyses producing a signal–noise ratio (S/N) of ≥3 and ≥ 10, respectively.

Assay precision, or repeatability, of GSH–NEM was determined by measuring the intra‐assay variation in the concentration of GSH–NEM from 13 replicates of a pooled plasma sample in a single run on the same day. The same process, but with 13 different replicates, was used to measure assay precision for GSSG. Assay accuracy, or reproducibility, of GSH–NEM was determined by measuring inter‐assay variation in the concentration of GSH–NEM in a pooled plasma sample on three different days. Five replicate samples of the pooled plasma were measured each day, for a total of 15 sample measurements. Assay accuracy for GSSG was determined via the same process, but with 15 samples derived from a different pooled plasma sample.

Analytical recovery, or percentage recovery, of GSH and GSSG was evaluated by calculating the recovery of GSH or GSSG spiked into individual or pooled plasma samples as follows:

$$\text{Recovery}\;\left(\%\right)=\left(\frac{\left(\text{measured concentration of spiked sample}\right)-\left(\text{measured concentration of unspiked sample}\;\right)}{\text{measured concentration of spiked buffer}}\right)\times 100\%$$

### 2.6 Analyte stability

The stability of GSH–NEM and GSSG in NEM‐treated plasma samples following storage at −80°C was examined at multiple time points ranging from 1 week to over 1 year after collection. Specifically, at 1, 12, 46 and 55 weeks post‐collection, frozen NEM‐treated plasma sample aliquots maintained at −80°C were allowed to thaw and equilibrate to RT. Each sample (100 μl) was transferred into a new microcentrifuge tube then processed as described in Section 2.3. GSH and GSSG levels in processed plasma extracts where determined following the analytical procedures previously described in Section 2.4.

### 2.7 Matrix effects

The post‐extraction addition method (King, Bonfiglio, Fernandez‐Metzler, Miller‐Stein, & Olah, 2000) was used to determine if the plasma sample extract induced a matrix effect (i.e. enhanced or suppressed analyte ionization) of GSH–NEM and GSSG. Integrated peak areas, or AUC, of ion abundance (i.e. ion counts min^−1^) of the analyte of interest (i.e. GSH–NEM and GSSG) at different concentrations were compared when prepared in assay buffer [i.e. 90% 0.1 M PB containing 5 mm NEM and 10% (v/v) TCA] vs.when they were prepared at the same concentration in plasma sample extract (i.e. sample supernatant recovered from a solution of 20% plasma, 70% 0.1 m PB containing 5 mm NEM and 10% TCA following protein precipitation and removal). Samples, calibration standards, controls and blanks were prepared in triplicate. The AUCs of GSH–NEM or GSSG in the standard solution were plotted as AUC values corresponding to the concentration of analyte, whereas the AUCs of GSH–NEM and GSSG in plasma sample extracts were plotted after first subtracting the AUC of a nonspiked plasma sample extract from the AUC of plasma sample extract spiked with either GSH–NEM or GSSG. Note that the AUCs of GSH–NEM or GSSG in standard solution or plasma sample extract were plotted following AUC normalization to an internal standard (i.e. either GSH–NEM–IS or GSSG–IS as appropriate). The slope of the line derived from the regression analysis of the triplicate datasets for GSH in buffer vs.GSH in plasma, or GSSG in buffer vs.GSSG in plasma, was then used to calculate the matrix effect based on the following equation (Taylor, 2005):

$$\text{Matrix effect}\;\left(\%\right)=100\%\times \left(1-\left\{\frac{\text{average slope of calibration curve in matrix}}{\text{average slope of calibration curve in assay buffer}}\right\}\right)$$

In order to determine if the matrix effect was statistically significant, the equal slopes test of ANCOVA was used to compare the slope of the regression line of the analyte of interest in plasma extract vs. the regression line of the analyte of interest in buffer. For each comparison, the matrix (i.e. buffer and plasma) was entered as the nominal variable, analyte concentration was entered as the covariate, and analyte AUC was entered as the dependent variable. A *P*‐value <0.05 for the equal slopes test indicated that the slopes of the regression lines were not equal, and that the plasma extract induced a matrix effect. Note that all comparisons satisfied the ANCOVA assumptions of homogeneity of variance and homogeneity of regression slopes.

## 3 RESULTS

### 3.1 Chromatography

**FIGURE 1:**
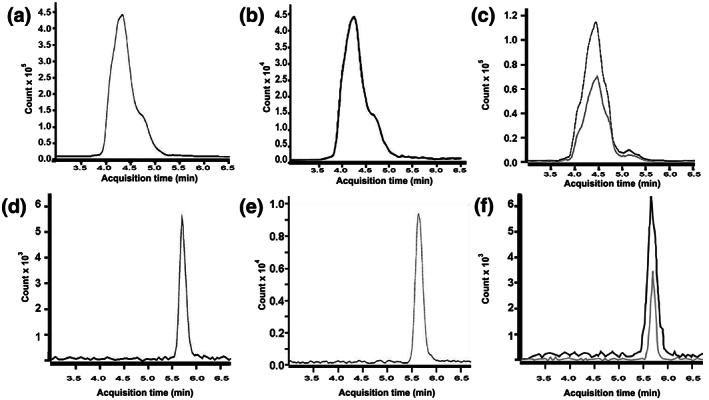
Representative LC–MS/MS multiple reaction monitoring chromatograms of: (a) 40 nmglutathione–*N*‐ethylmaleimide(GSH–NEM) in buffer; (b) 20 nmGSH–NEM–internal standard (IS) in buffer; (c) GSH–NEM in plasma before (gray trace) and after (black trace) spiking with 40 nmGSH–NEM; (d) 20 nm glutathione disulfide (GSSG) in buffer; (e) 20 nmGSSG–IS in buffer; and (f) GSSG in plasma before (gray trace) and after (black trace) spiking with 20 nm GSSG

Typical LC–MS/MS chromatograms of GSH–NEM, GSH–NEM–IS, GSSG and GSSG–IS are shown in Figure 1. The retention time of GSH–NEM (Figure 1a) and GSH–NEM–IS (Figure 1b) in buffer was 4.2 min. The retention time for GSSG (Figure 1d) and GSSG–IS (Figure 1e) in buffer was 5.8 min. Note that the retention times of the analyte of interest and its corresponding internal standard are identical. Representative examples of the GSH–NEM and GSSG LC–MS/MS response in deproteinized human plasma samples are provided in Figure 1c and f, respectively. Chromatograms of both analyte‐spiked and unspiked samples are displayed together in the same plot area as black and gray traces, respectively.

### 3.2 Method validation

Data for assay specificity are presented in Table 1. The mean retention time of the GSH–NEM quantifier ion in buffer was 253.4 s (4.223 min). The minimum and maximum retention times of the GSH–NEM quantifier ion in plasma were 251.8 and 257.1 s (4.197–4.285 min), respectively. For GSSG, the mean retention time of the quantifier ion was 347.5 s (5.792 min) in buffer, compared with minimum and maximum retention times of 345.2 and 348.6 s (5.753–5.810 min) in plasma. Note that for both GSH–NEM and GSSG, the minimum and maximum retention times of the quantifier ion in plasma were within ±6 s (±0.1 min) of the mean retention time of the quantifier ion in buffer. The ratio of qualifier–quantifier ions for GSH–NEM and GSSG is also presented in Table 1. A comparison of qualifier–quantifier ion ratios in plasma vs. the corresponding qualifier–quantifier ion ratios in buffer demonstrates that the accuracy tolerance reached a maximum value of 18.6% for the GSH–NEM*m*/*z* 113:158.1 ion ratio. All other accuracy tolerance values were <4%, and none exceeded ±20%. Note that quantifier ion retention time and qualifier–quantifier ion ratios are criteria for the validation of method specificity, and the values of ±6 s for retention time and ±20% for accuracy tolerance are considered as the maximum acceptable deviations in analytical LC–MS/MS methods (US Food and Drug Administration, 2018).

**TABLE 1:** Analyte retention time, quantifier: qualifier ion ratios and accuracy tolerance for GSH–NEM and GSSG in buffer and plasma^a^

Analyte	Matrix	Retention time (s)	Quantifier ion	Qualifier ion 1	Qualifier ion 2	Qualifier–quantifier ion ratios	
			(*m*/*z* 158.1)	(*m*/*z* 113.0)	(*m*/*z* 84.1)	(113.0:158.1)	(84.1:158.1)
GSH^b^	Buffer	253.4 (251.8–254.6)	1789 (1629–2137)	1001 (843–1314)	783 (717–935)	55.6 ± 5.0%	43.8 ± 0.9%
	Plasma	255.0 (251.8–257.1)	653 (570–831)	444 (402–534)	297 (265–382)	68.3 ± 3.0%	45.5 ± 1.1%
Accuracy tolerance^c^						18.6%	3.74%
			(*m*/*z* 231.1)	(*m*/*z* 176.7)	(*m*/*z* 178.7)	(176.7: 231.1)	(178.7: 231.1)
GSSG^d^	Buffer	347.5 (345.4–348.5)	11,337 (10,021–12,767)	5813 (5203–6791)	1507 (1189–1798)	51.4% ± 3.13%	13.3% ± 1.71%
	Plasma	346.2 (345.2–348.6)	1985 (1647–2384)	986 (852–1141)	271 (210–371)	51.1% ± 13.8%	13.5% ± 1.98%
Accuracy tolerance^c^						−0.58%	1.61%

a Data are presented as mean (min − max) or mean ± SD, *n* = 4 per group

b Values are area under the curve (AUC) × 10^−3^.

c Accuracy tolerance = [1 − (mean buffer qualifier–quantifier ratio/mean plasma qualifier–quantifier ratio)] × 100%.

d Values are peak height.

GSH, Glutathione; GSSG, glutathione disulfide; NEM, *N*‐ethylmaleimide.

**FIGURE 2:**
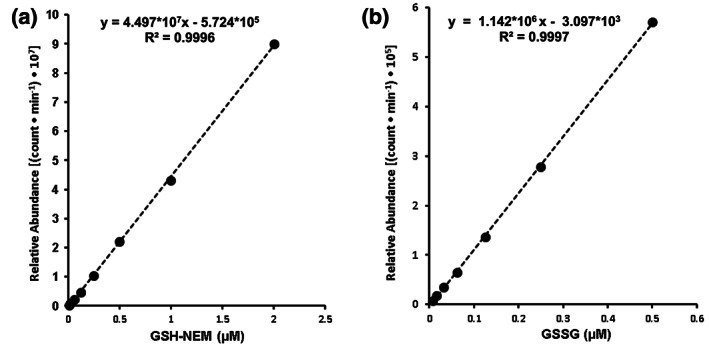
Calibration curves displaying linear responses of (a) GSH–NEM and (b) GSSG

Glutathione LC–MS/MS assay linearity was examined from ~16 nm to 2 μm for GSH–NEM analysis and from ~2 to 500 nm for GSSG analysis. Standard calibration curves, regression equations and coefficients of determination (*R* ^2^) for GSH and GSSG LC–MS/MS analysis are displayed in Figure 2a and b, respectively. Calibration standards provided a linear response from 16 nm to 2 μm for GSH–NEM and from 2 nm to 500 nm for GSSG. The LLOD, defined as the lowest standard concentration resulting in S/N ≥ 3, was 0.98 nm for GSH–NEM and 0.65 nm for GSSG. The LLOQ, the lowest concentration yielding a S/N ≥ 10, was 4.99 nm for GSH–NEM and 3.65 nm for GSSG.

Assay precision, as determined by assay repeatability (intra‐assay variation), and assay accuracy, as determined by assay reproducibility (inter‐assay variation), are presented in Table 2. Intra‐assay variation, expressed as the coefficient of variation (CV), was 3.7% for GSH–NEM and 1.9% for GSSG. The CV(%) for reproducibility (inter‐assay variation) was 7% for GSH–NEM and 2.8% for GSSG. All values were within the ±15 CV(%) limit defined by the US Food and Drug Administration (2018).

**TABLE 2:** Intra‐ and inter‐assay variation of GSH and GSSG in a pooled NEM plasma sample analyzed 13 times within one day (intra‐assay) or five times daily for 3 days (inter‐assay)

	Intra‐assay (*n* = 13)			Inter‐assay (*n* = 15)		
	Average (μm)	Standard deviation (μm)	CV (%)	Average (μm)	Standard deviation (μm)	CV (%)
GSH–NEM	1.0267	0.0378	3.7%	1.193	0.0840	7.0%
GSSG	0.1220	0.0023	1.9%	0.0891	0.0025	2.8%

Recovery of GSH and GSSG was determined by comparing the measured concentration of each analyte in plasma before and after spiking with a known, physiologically relevant quantity of GSH or GSSG (Table 3). The average GSH–NEM spike recovery was 98.0 ± 7.64%, whereas the average spike recovery of GSSG was 98.5 ± 12.7%. Table 4 summarizes the long‐term stability of GSH–NEM and GSSG when stored at −80°C. GSH–NEM remained remarkably stable throughout the 55‐week storage period, as evidenced by the averages of 7.6, 12.3 and 13.1% CV at weeks 12, 46 and 55, respectively. GSSG exhibited remarkable stability throughout the 46‐week storage period reflected by average values of CV of 8.6% at week 12 and 9.6% at week 46. GSSG stability in NEM‐treated plasma stored at −80°C appeared to decline significantly between week 46 and week 55, as suggested by an average CV of 27.2%. Note that, although there was insufficient sample to quantify GSSG in two subjects (i.e. S1 and S2) at week 55, this level of variability (CV) indicates that the current sample preservation method may not be satisfactory for GSSG analysis of samples stored longer than 46 weeks.

**TABLE 3:** Analytical recovery of GSH and GSSG spiked into plasma samples containing 5 mm NEM

Spiked compound (thiol status)	Internal standard (isotope)	[Spiked compound]^a^	[Spiked compound]^a^ measured	Recovery spiked compound (%)^d^
GSH (free thiol)^b^ ^,^ ^c^	GSH‐d5–NEM	1.0 μm	0.98 ± 0.08 μm	98.0 ± 7.64%
GSSG^b^	GSSG‐^13^C_4_^15^N_2_	20 nm	19.7 ± 2.53 nm	98.5 ± 12.7%

a Indicates final concentration in plasma.

b For GSH (free thiol), *n* = 3 independent pooled plasma samples; for GSSG, *n* = 5 independent samples.

c Note GSH was spiked into plasma as the free thiol, but quantified by LC–MS/MS as the GSH–NEM conjugate.

d Spike recovery (%) = 100% × (spiked compound measured in sample/spiked compound measured in assay buffer).

**TABLE 4:** Stability of GSH–NEM and GSSG in samples stored at −80°C for 1–55 weeks after collection

	Subject/sample	Analyte (μm) measured in samples kept at −80°C for:				Average concentration (μm) over 55 weeks	Percentage of initial analyte measured at:			Cumulative CV(%) at different time points:		
		1 week	12 weeks	46 weeks	55 weeks		12 week	46 weeks	55 weeks	12 week	46 weeks	55 weeks
GSH–NEM	S1	1.682	1.781	2.725	2.794	2.245	105.9%	162.0%	166.2%	4.0%	27.9%	26.5%
	S2	1.457	1.856	1.531	1.398	1.561	127.4%	105.1%	96.0%	17.0%	13.1%	13.1%
	S3	2.350	2.225	1.991	2.093	2.165	94.7%	84.7%	89.1%	3.9%	8.3%	7.2%
	S4	1.156	1.082	1.358	1.478	1.269	93.6%	117.4%	127.8%	4.7%	11.9%	14.3%
	S5	2.277	1.982	2.184	2.261	2.176	87.1%	95.9%	99.3%	9.8%	7.0%	6.2%
	Pool	1.547	1.686	1.532	1.932	1.674	109.0%	99.0%	124.9%	6.1%	5.4%	11.1%
	Average						102.9%	110.7%	117.2%	7.6%	12.3%	13.1%
GSSG	S1	0.064	0.058	0.048	‐	0.057	91%	74%	‐	7%	15%	15%
	S2	0.030	0.035	0.031	‐	0.032	115%	104%	‐	10%	7%	7%
	S3	0.075	0.086	0.079	0.124	0.091	115%	106%	166%	10%	7%	25%
	S4	0.035	0.040	0.042	0.098	0.054	116%	121%	282%	10%	10%	55%
	S5	0.088	0.072	0.086	0.151	0.099	82%	99%	172%	14%	11%	36%
	Pool	0.057	0.056	0.067	0.090	0.067	99%	118%	159%	1%	10%	24%
	Average						103%	104%	195%	9%	10%	29%

Stability of GSH–NEM and GSSG was also evaluated in a subset of samples stored at 4°C. After 1 week of storage at this temperature GSH–NEM concentration in all samples was <9% of aliquots of the same samples stored at −80°C and GSSG was below the detectable limit. By 12 weeks GSH–NEM and GSSG levels in all samples stored at 4°C were not detectable using the procedures detailed in this report (data not shown). Based on these observations, it is recommended that samples be stored at −80°C to maximize preservation of target analytes.

### 3.3 Matrix effects

**FIGURE 3:**
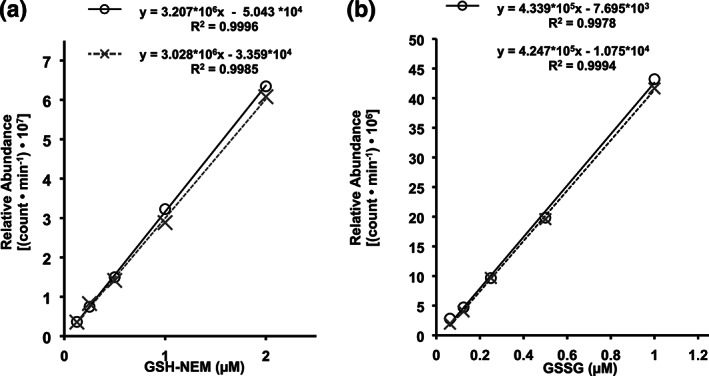
Matrix effect. The effect of the trichloroacetic acid‐deproteinized plasma sample extract matrix (dashed lines) on the LC–MS/MS responses of GSH (a) and GSSG (b) compared with responses in standard buffer preparations (solid lines)

There was a significant matrix effect of the plasma sample extract on the ionization intensity of GSH, but not GSSG (Figure 3). Regression analysis (i.e. slope of the line) indicated that the ionization intensity (AUC) of GSH–NEM decreased by ~5.6% when evaluated in plasma matrix compared with buffer (Figure 3a). This decline was statistically significant (ANCOVA, *P* = 0.02). In contrast, GSSG ionization intensity was not affected by the plasma matrix. The regression slope of GSSG in plasma was reduced by a nonsignificant (ANCOVA, *P* = 0.432) ~2.1% compared with the regression slope of GSSG in buffer.

## 4 DISCUSSION

Early assessments of GSH and GSSG used techniques that did not address a number of factors now known to introduce artifactual interference (Rossi et al., 2006). As such, the validity of data derived from these assays is open to question. However, more recent developments concerning sample collection and processing as well as storage and handling techniques have dramatically improved the reliability of GSH and GSSG measurements, particularly as an indicator of oxidative stress in various types of biological samples (Giustarini et al., 2011; Rossi et al., 2006). To date, ongoing research in several laboratories has established multiple strategies for GSH and GSSG determination in human whole blood and isolated RBCs that are effective (Giustarini et al., 2013; Rossi et al., 2006; Squellerio et al., 2012); however, these methods involve intensive collection and processing procedures and require proficiency in difficult/complex techniques to ensure accurate measurements. Furthermore, many of the methods that perform well with human whole‐blood samples and RBC lysates do not translate well for evaluation of GSH and GSSG in human plasma samples (Giustarini et al., 2013). In the current report, we describe a new approach specifically developed to address a wide range of problems that have historically contributed to erroneous estimates of GSH and GSSG in human plasma. In this specialized procedure a novel LC–MS/MS method was developed to examine endogenous concentrations of the two major glutathione redox forms in clinical plasma samples acquired and handled using simplified collection and processing procedures (see Materials and Methods). The methods described in this report are straightforward, can be executed in comparatively short periods of time and moreover provide accurate estimates of endogenous GSH and GSSG levels in human plasma samples that correspond to previously published values derived from methodologies that are considerably more challenging technically than the method presented herein (Claeson et al., 2019; Jones et al., 1998; Jones & Liang, 2009). As such, this technique can be applied in array settings that have not previously been available for human clinical research.

Thiol moieties are highly unstable. It is therefore unsurprising that GSH and GSSG readily undergo biochemical changes both *in vivo* and *ex vivo.* To address the potential influence of *ex vivo* thiol oxidation and reactivity on measurements of GSH and GSSG, we enacted three specific steps. First, we selected NEM as a protective thiol‐masking reagent to prevent artifactual GSH oxidation. Indeed, for whole‐blood glutathione analyses, NEM is typically added directly to the sample at the time of collection and is often added to collection tubes before drawing blood samples (Giustarini et al., 2013). However, NEM exhibits significant hemolytic activity (Kuypers et al., 1996). Therefore, in order to avoid potential contamination of plasma with RBC‐derived GSH and GSSG, reported to be orders of magnitude higher in RBCs than in plasma (Giustarini et al., 2013), the separation of plasma from whole blood samples was performed in the absence of NEM at refrigeration temperatures (i.e. 2–4°C) over a brief period of time (~12 min). Once collected, plasma supernatant was transferred directly into tubes prefilled with NEM. Second, as an additional measure to prevent *ex vivo* changes in glutathione oxidation states, we collected blood samples in vacutainers containing EDTA, a well‐established chelating agent (Cotton, 2003). This was done to reduce the potential for transition‐metal catalyzed GSH oxidation which is known to occur in whole blood (Squellerio et al., 2012). Finally, because GSH displays high instability at room temperature, particularly in the absence of protective reagent or preservation buffers (Claeson et al., 2019), we processed all samples on ice or via refrigerated centrifugation (4°C), as soon as the blood draw was completed. The combined effect of each of these steps was to maintain the integrity of plasma GSH and GSSG levels, as evidenced in particular by the extremely low levels of plasma GSSG (i.e. < 100 nm) measured via this method that are as low as or lower than recently reported values (Bettermann et al., 2018; Claeson et al., 2019).

In addition to thiol reactivity, we identified two other key issues that were critical in establishing this method of GSH and GSSG analysis in human plasma. The first was buffer pH; previous research from other laboratories provides evidence that alkaline conditions may facilitate hydrolysis of GSH–NEM adducts at multiple positions, potentially generating products with different mass fragments and ionization states that could confound interpretations of LC–MS/MS analyses (Nishiyama & Kuninori, 1992; Roosild et al., 2010). Evidence suggesting the potential influence this may have on our measurements of GSH and GSSG includes observations that detection of GSH–NEM adduct was exclusively achieved when conditions were kept slightly acidic to acidic during sample preparation (data not published). We also found that ionization of GSH and GSSG was sensitive to reagents and procedures used in sample preparation and processing. For example, changing the extraction agent from TCA to MPA perturbed ionization of analytes and limited detection to a range that was insufficient for quantification of GSH and GSSG in plasma samples, even with minimal sample dilution. In addition, we found that adjusting the concentration of TCA from 10% w/v to 5% w/v: (a) exacerbated chromatography problems as a consequence of incomplete protein precipitation; and (b) significantly reduced GSH–NEM and GSSG ionization during LC–MS/MS analyses. However, the use of 10% TCA optimized both protein precipitation and GSH–NEM adduct formation and, moreover, had negligible effects on analyte ionization properties as evidenced by a minimal (i.e. ~5.6%) and nonexistent matrix effect of the plasma extract on GSH–NEM and GSSG, respectively.

A number of recent publications have detailed approaches that simultaneously measure GSH and GSSG (Bondada et al., 2016; Guan, Hoffman, Dwivedi, & Matthees, 2003; Harwood, Kettle, Brennan, & Winterbourn, 2009). Although these procedures may be applicable to measuring GSH and GSSG in many types of samples, they are inadequate for the simultaneous measurement of GSH and GSSG in human plasma for several reasons. In particular, many of these methods lack the sensitivity necessary to measure GSH and GSSG in plasma, which requires LLOQs in the nanomolar to subnanomolar range. Indeed, after failing to achieve appropriate sensitivity (i.e. LLOD and LLOQ) using previously published methods that were designed for the simultaneous detection of GSH and GSSG, we adjusted our strategy to employ two separate protocols, one to measure each redox form of glutathione. By separating different analytes on separate LC columns with different mobile phases we were able to detect GSH and GSSG as distinct, well‐defined, integrated peaks on individual chromatograms (Figure 1), improving sensitivity to meet the necessary range for clinical plasma analyses. Although this approach required two distinct preparations and two distinct LC–MS/MS analytical conditions in order to measure GSH and GSSG in each sample, the superior resolution of the distinct procedures markedly improved GSH and GSSG detection sensitivity, with assay LLODs in the nanomolar and subnanomolar range, respectively. In addition, performing two different procedures did not require an excessive investment of time as sample processing and preparation could be performed rapidly (about 2–3 h to prepare 20–30 samples) and the run‐time for each injection was completed within 20 min for GSH and in <10 min for GSSG. Note that for GSH–NEM the run time (20 min) was considerably longer than the retention time (4.2 min). This run time was selected empirically as a precaution to ensure removal of nontarget substances from the LC–column matrix that might otherwise interfere with or obscure the precise detection and quantification of GSH–NEM in subsequent samples. Indeed, we did not observe any sample carryover or contamination throughout the duration of this study, and attribute this in part to the extended GSH–NEM run time.

A recent study by Claeson et al. described an ultra‐performance–LC–MS/MS (UPLC–ESI–MS/MS) approach for measuring GSH and GSSG in human plasma (Claeson et al., 2019). Although the method is well suited for investigating the impact of preparation and storage conditions on reduced and oxidized levels of glutathione with a reported linear range between 0.1 and 10 μm for both GSH and GSSG, this sensitivity is not adequate for identifying subtle changes in clinical treatment paradigms. In addition, the method does not involve precautionary measures to protect samples from auto‐oxidation and the authors report notable shifts in analyte detection after 3 months of storage at −80°C. The approach detailed in this report includes simple steps to maintain analyte stability, which remains apparent over ~1 year of storage (as shown in Table 3), and allows detection in a linear range from 2 to 500 nm for GSSG and from 16 nm to 2 μm for GSH.

## 5 CONCLUSION

In conclusion, the processes and procedures we have detailed in the current work provide a highly sensitive, yet simple and rapid method for evaluation of GSH and GSSG in human plasma samples with a wide range of advantages compared with previous approaches performed alone. The detection range and stability we established for these procedures indicate that this method can identify minimal variations in plasma GSH and GSSG levels that could not be discriminated via previous methods.

## CONFLICTS OF INTEREST STATEMENT

All authors were employed by USANA Health Sciences, Inc.

## FUNDING INFORMATION

USANA Health Sciences Inc. was the sole provider of funding and research support.
